# The m6A Reader IGF2BP2 Regulates Macrophage Phenotypic Activation and Inflammatory Diseases by Stabilizing TSC1 and PPAR*γ*

**DOI:** 10.1002/advs.202100209

**Published:** 2021-05-03

**Authors:** Xia Wang, Yuge Ji, Panpan Feng, Rucheng Liu, Guosheng Li, Junjie Zheng, Yaqiang Xue, Yaxun Wei, Chunyan Ji, Dawei Chen, Jingxin Li

**Affiliations:** ^1^ Department of Physiology School of Basic Medical Sciences Cheeloo College of Medicine Shandong University Jinan Shandong 250012 China; ^2^ Department of Hematology Qilu Hospital Cheeloo College of Medicine Shandong University Jinan Shandong 250012 China; ^3^ ABLife BioBigData Institute Wuhan Hubei 430075 China; ^4^ Center for Genoem Analysis ABLife Inc. Wuhan Hubei 430075 China; ^5^ Laboratory of Medical Chemistry Interdisciplinary Cluster for Applied Genoproteomics (GIGA) Stem Cells University of Liège CHU, Sart‐Tilman Liège 4000 Belgium

**Keywords:** IGF2BP2, inflammatory diseases, m^6^A reader, macrophage polarization, TSC1

## Abstract

Phenotypic polarization of macrophages is regulated by a milieu of cues in the local tissue microenvironment. Currently, little is known about how the intrinsic regulators modulate proinflammatory (M1) versus prohealing (M2) macrophages activation. Here, it is observed that insulin‐like growth factor 2 messenger RNA (mRNA)‐binding protein 2 (IGF2BP2)‐deleted macrophages exhibit enhanced M1 phenotype and promote dextran sulfate sodium induced colitis development. However, the IGF2BP2^−/−^ macrophages are refractory to interleukin‐4 (IL‐4) induced activation and alleviate cockroach extract induced pulmonary allergic inflammation. Molecular studies indicate that IGF2BP2 switches M1 macrophages to M2 activation by targeting tuberous sclerosis 1 via an N6‐methyladenosine (m^6^A)‐dependent manner. Additionally, it is also shown a signal transducer and activators of transcription 6 (STAT6)‐high mobility group AT‐hook 2‐IGF2BP2‐peroxisome proliferator activated receptor‐*γ* axis involves in M2 macrophages differentiation. These findings highlight a key role of IGF2BP2 in regulation of macrophages activation and imply a potential therapeutic target of macrophages in the inflammatory diseases.

## Graphical abstract

Insulin‐like growth factor 2 mRNA‐binding protein 2 (IGF2BP2) switches M1 macrophages to M2 activation by targeting tuberous sclerosis 1 and peroxisome proliferator activated receptor‐*γ* via an N6‐methyladenosine‐dependent manner. Myeloid depletion of IGF2BP2 promotes dextran sulfate sodium induced colitis development while alleviates cockroach extract induced pulmonary allergic inflammation. These findings imply a potential therapeutic target of macrophages in the inflammatory diseases.

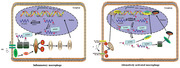

## 1 Introduction

Macrophages play a dynamic role in the control of the tissue homeostasis, initiation of inflammation and modulation of inflammatory resolution.^[^ ^1^ ^]^ It is broadly believed that macrophages are activated by multiple signals and traditionally classified into two distinct subtypes: classically activated (M1) macrophages and alternatively activated (M2) macrophages.^[^ ^2^ ^]^ Inflammatory macrophages, which are activated by toll‐like receptors (TLR) ligands and interferon *γ* (IFN‐*γ*), produce proinflammatory cytokines and induce a Th1 response to mediate host defense against infections and tumor cells.^[^ ^1^ ^]^ Anti‐inflammatory macrophages are induced by interleukin‐4 (IL‐4), interleukin‐10 (IL‐10), interleukin‐13 (IL‐13), glucocorticoids or immune complexes, promote Th2 immunity and regulate wound healing and tissue remodeling.^[^ ^3^ ^]^ In tumors, the tumor associated macrophages (TAMs), which are usually triggered by environmental cues to exhibit an M2 similar phenotype, contribute to build an immunosuppressive tumor niche ^[^ ^4^ ^]^ Although extracellular signals that induce macrophage polarization are well recognized,^[^ ^5^ ^]^ the intrinsic regulators, which shapes macrophage phenotype, have not been well investigated.

The mammalian target of rapamycin (mTOR) signaling pathway senses both intracellular and extracellular signals and serves as a regulator of the fate of immune cell populations.^[^ ^6^ ^]^ The tuberous sclerosis (TSC) complex comprising TSC1 and TSC2 inhibits Rheb small GTPase directly, and Rheb is the upstream of the rapamycin‐sensitive complex 1 (mTORC1), thus to downregulate mTORC1 activity.^[^ ^7^ ^]^ Genetic loss of TSC1 increased proinflammatory response in macrophages while highly resist to IL4 induced M2 polarization.^[^ ^8^ ^]^ Activation of mTORC1 accelerates the synthesis of fatty acids and cholesterol though the transcriptional factor peroxisome proliferator activated receptor‐*γ* (PPAR*γ*), which promotes the metabolic reprograming in the anti‐inflammatory macrophages.^[^ ^9^ ^]^ While the activation of mTORC1 is relatively well understood at the level of nutrients, growth factors and signal transduction, the post‐transcriptional regulation of the component expression in the mTORC1 pathway remain less clear.

It is well known that post‐transcriptional modifications of messenger RNAs (mRNAs), among which m^6^A is the most abundant internal RNA modification, regulate genes expression by influencing mRNA splicing, stability, translocation and translation.^[^ ^10^ ^]^ m^6^A modification is deposited by “writers” the methyltransferase complex containing the methyltransferase‐like 3 and 14 proteins (METTL3 and METTL14) and their regulator Wilms tumor 1‐associated protein, and removed by “erasers” demethylases: fat mass and obesity‐associated protein (FTO) and α‐ketoglutarate dependent dioxygenase AlkB homolog 5 (ALKBH5).^[^ ^11^, ^12^, ^13^, ^14^ ^]^ Moreover, m^6^A modification exerts its biological functions by “readers”: YTH (YT521‐B homology) domain proteins including YTHDC1–2 and the YTH‐family proteins YTHDF1–3 as well as insulin‐like growth factor 2 mRNA binding proteins IGF2BP1–3.^[^ ^15^ ^]^ Among these m^6^A readers, IGF2BP2 binds RNA via its six characteristic RNA‐binding domains, containing two RNA recognition motifs (RRM1 and RRM2) and four K Homology (KH) domains (KH1–KH4).^[^ ^16^ ^]^ Dysregulation of IGF2BP2 is implicated in certain diseases such as diabetes and cancer, nevertheless, little is known about IGF2BP2's functions in immunity.

Here, we found that IGF2BP2 is highly expressed in both of M1 and M2 macrophages, IGF2BP2^–/–^ macrophages are hyporesponsive to IL‐4 whereas the inflammatory response to lipopolysaccharides (LPS) was enhanced both in culture and in vivo. Mechanistically, IGF2BP2 skewed M1 macrophages to M2 activation via TSC1‐mTORC1 pathway and PPAR*γ* mediated fatty acids uptake. Additionally, our data elucidate that IGF2BP2 binds to TSC1 and PPAR*γ* directly and regulates TSC1 and PPAR*γ* expression by serving as an m^6^A reader.

## 2 Results

### 2.1 Deletion of IGF2BP2 Potentiates M1‐Like Macrophage Polarization

**Figure 1:**
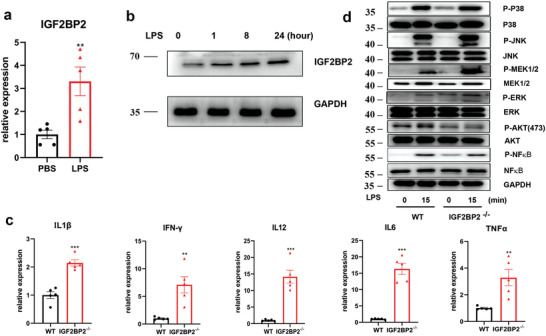
IGF2BP2‐deficient BMDMs are hypersensitive to LPS. a) IGF2BP2 mRNA expression in BMDMs after LPS treatment for 24 h; *n* = 5. b) IGF2BP2 protein expression in BMDMs after LPS treatment at indicated time points; *n* = 4. c) Expression of IL1*β*, IFN‐*γ*, IL12, IL6, and TNF‐α in the WT and IGF2BP2 deficient BMDMs were determined by real‐time PCR after LPS stimulation for 24 h; *n* = 5. d) The activity of p38 MAPK, ERK, JNK, MEK, AKT, and NF‐*κ*B in both WT and IGF2BP2^–/–^ BMDMs were detected by western blotting after LPS treatment at indicated time points; *n* = 5. Data were shown as mean ± SEM. **p < 0.01, ***p < 0.001 versus the phosphate buffered saline (PBS) or WT group by two‐tailed unpaired Student's *t*‐test

We first determined the expressions of IGF2BP1, IGF2BP2, and IGF2BP3 in macrophages. Bone marrow‐derived macrophages (BMDMs) (**Figure** **1**a,b) and peritoneal macrophages (PMs) (Figure S1b,c, Supporting Information) expressed higher mRNA and protein levels (Figure S6a,b, Supporting Information) of IGF2BP2, whereas IGF2BP3 mRNA did not change after LPS stimulation (Figure S1a, Supporting Information), and IGF2BP1 was not expressed in macrophages. To address whether IGF2BP2 play a role during macrophage M1 polarization, we generated BMDMs and peritoneal exudate cells (PECs) from wild‐type (WT) and IGF2BP2^–/–^ mice. After LPS stimulation for 24 h, IGF2BP2^–/–^ BMDMs exhibited substantially increased expression of IL1*β*, IFN‐*γ*, IL12, IL6, and tumor necrosis factor (TNF)‐α (Figure 1c), similar upregulated cytokines were seen in PMs (Figure S1d, Supporting Information), as determined by real‐time quantitative reverse transcription PCR (qRT‐PCR). LPS can provoke several signal pathways including p38 mitogen‐activated protein kinase (MAPK), nuclear factor kappa‐light‐chain‐enhancer of activated B cells (NF‐*κ*B), c‐Jun N‐terminal kinases (JNK), MEK‐ERK, and phosphatidilinositol‐3 kinase (PI3K)/Protein kinase B (AKT) cascade, as well as signal transducers and activators of transcription signals in M1 macrophages.^[^ ^17^, ^18^, ^19^ ^]^ Established on these researches, we analyzed the status of correlative signal pathway in both WT and IGF2BP2 deficient BMDMs by western blotting (Figure 1d and Figure S6e, Supporting Information). MEK1/2 and ERK activities were augmented in IGF2BP2 deficient BMDMs compared with WT BMDMs after LPS stimulation (Figure 1d and Figure S6e, Supporting Information), whereas IGF2BP2‐deficient BMDMs showed equal levels of p‐p38 MAPK, p‐NF‐*κ*B, phosphorylated signal transducer and activators of transcription 1 (p‐STAT1) (Figure S1e, Supporting Information), phosphorylated c‐Jun N‐terminal kinases (p‐JNK) as WT macrophages. In contrast, IGF2BP2^–/–^ BMDMs expressed faint p‐AKT (Ser473) activation compared with WT after LPS treatment (Figure 1d and Figure S6e, Supporting Information). However, the results suggested that deletion of IGF2BP2 had no significant effect on transcriptional level (Figure S1f, Supporting Information). Collectively, our data indicate that loss of IGF2BP2 could enhance M1‐like macrophage phenotype.

### 2.2 STAT6 Binds Directly to High Mobility Group AT‐Hook 2 (HMGA2) Mediated IGF2BP2 Expression in Response to IL‐4 and IGF2BP2 Deficiency Impairs M2 Activation

**Figure 2:**
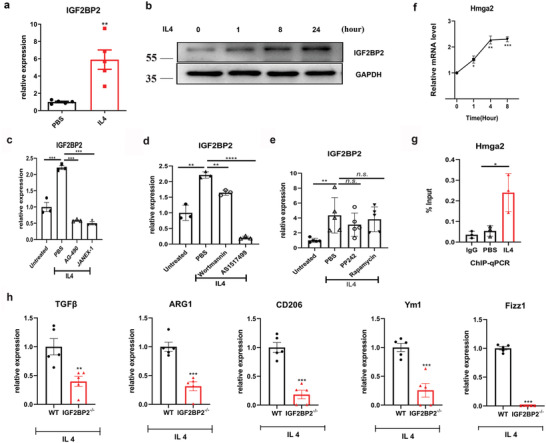
IL‐4 acts through JAK‐STAT6 pathway to induce IGF2BP2 expression and IGF2BP2 deficiency impaired M2 polarization. a) IGF2BP2 mRNA expression in BMDMs after IL4 treatment for 24 h; *n* = 5. b) IGF2BP2 protein expression in BMDMs after IL4 treatment at indicated time points; *n* = 4. c–e) BMDMs nonactivated or alternatively activated (IL‐4) macrophages were exposed to PBS, c) AG‐490 (80 µmol), JANEX‐1 (80 µmol), d) Wortmannin (200 nmol), AS1517499 (5 µmol), e) PP242 (200 nmol) or Rapamycin (40 nmol) for 8 h; c,d) *n* = 3 or e) *n* = 5. f) Hmga2 mRNA expression in BMDMs after IL4 treatment at indicated time points; *n* = 3. g) ChIP analyses of the binding efficiency of STAT6 to the Hmga2 gene promoter in BMDMs with PBS or IL4 treatment for 24 h; *n* = 3. h) Expressions of ARG1, Ym1, Fizz1, CD206, and TGF‐*β* in the WT and IGF2BP2^–/–^ BMDMs were determined by real‐time PCR after IL4 stimulation for 24 h; *n* = 5. Data were shown as mean ± SEM. n.s.: no significant, **p < 0.01, ***p < 0.001 versus the PBS or WT group. *P*‐values were determined by using a,f,g,h) unpaired, two‐tailed Student's *t*‐test or c,d,e) two‐way analysis of variance (ANOVA).

Because loss of IGF2BP2 potentiates M1‐like phenotype, we considered that IGF2BP2 could play additional roles in control of M2 activation and then turned to an analysis of M2 related genes. In agreement with the results in LPS, IGF2BP2 was dramatically increased in BMDMs (**Figure** **2**a,b and Figure S6c, Supporting Information) and PMs (Figures S2b,c and S6d, Supporting Information) and no difference of IGF2BP3 mRNA after IL4 stimulation (Figure S2a, Supporting Information). Predictably, IGF2BP2^–/–^ BMDMs and PMs exhibited substantially reduced mRNA expression of Arg1, CD206, Ym1, Fizz1, and transforming growth factor beta (TGF‐*β*) as determined by qRT‐PCR (Figure 2h and Figure S2d, Supporting Information) or protein levels of programmed cell death 1 ligand 2 (PD‐L2) and RELMα (Figure S2e,f, Supporting Information).

IL‐4R signaling leads to activation of janus kinase (JAK) and downstream phosphorylation of STAT6 as well as PI3K‐Akt‐mTORC1 signaling in macrophages inducting anti‐inflammatory macrophage polarization.^[^ ^8^, ^20^ ^]^ To identify which downstream effectors of IL‐4 impact IGF2BP2 expression and/or activity, we used pharmacological inhibitors. JAK2 inhibitor AG‐490, JAK3 inhibitor JANEX‐1 or PI3K inhibitor Wortmannin, signal transducer and STAT6 inhibitor AS1517499, inhibited upregulated of an IL‐4‐regulated IGF2BP2 gene, implying that these effects of IL‐4 are mediated by JAK/STAT6 pathway (Figure 2c,d). Whereas, we found that blocking mTOR signaling with mTOR inhibitor PP242 or mTORC1 inhibitor Rapamycin failed to inhibit the enhanced IGF2BP2 production after IL4 treatment (Figure 2e) implying that IL‐4 regulated IGF2BP2 activity via JAK‐STAT6 pathway rather than PI3K‐mTOR pathway. Importantly, HMGA2, a member of the HMGA gene family, regulates IGF2BP2 transcription during embryonic development and myoblast proliferation and myogenesis.^[^ ^21^, ^22^ ^]^ Similar to the observations in IGF2BP2 changes (Figure 2a), we detected significantly increased levels of HMGA2 with IL4 treatment (Figure 2f). To further confirm whether STAT6 directly binds to HMGA2 and modulates expression of IGF2BP2, we performed chromatin immunoprecipitation (ChIP) assays in BMDMs. The results proved that STAT6 directly bound to the promoter region of HMGA2 in the presence of IL4 (Figure 2g). Taken together, these findings strongly indicated that STAT6 bound to HMGA2 promoter and promoted IGF2BP2 expression to regulate IL‐4 induced M2 macrophage activation.

### 2.3 IGF2BP2 Selectively Binds a Subset of mRNAs Involved in Macrophage Functions

To explore the mechanism how IGF2BP2 regulates M2 macrophages activation, we obtained a transcriptome‐wide binding profile of IGF2BP2 in macrophages by employing an improved RNA immunoprecipitation‐coupled high‐throughput sequencing (iRIP‐seq) approach, a recently developed UV‐crosslinking immunoprecipitation method.^[^ ^23^ ^]^ Displaying the RNA‐seq data in a volcano plot (Figure S3a, Supporting Information) and using a log2 fold‐change cutoff of 2.0 and a *P*‐value cutoff of 0.05, we defined 787 genes bound by IGF2BP2 (Table S3, Supporting Information). Among these top enriched genes, we noticed that several genes (Kcnn4, Arg1, Hist1h1b, Hist1h4c, Cd74, Ak2, and H2‐Eb1) are associated with M2 macrophage polarization.^[^ ^24^ ^]^ And macrophage colony‐stimulating factor (M‐CSF) is essential to the regulation of mTORC2 signaling in M2 macrophages,^[^ ^25^ ^]^ it provides a high possibility that regulation of M‐CSF receptor (Csf1r) expression by IGF2BP2 might contribute to M2 macrophages activation Furthermore, the gene ontology (GO) analysis of mammalian phenotype ontology showed that these genes involved in abnormal macrophage physiology and phagocyte morphology (Figure S3b, Supporting Information).

### 2.4 IGF2BP2 Stabilizes Methylated PPAR*γ* mRNA in Macrophages

**Figure 3:**
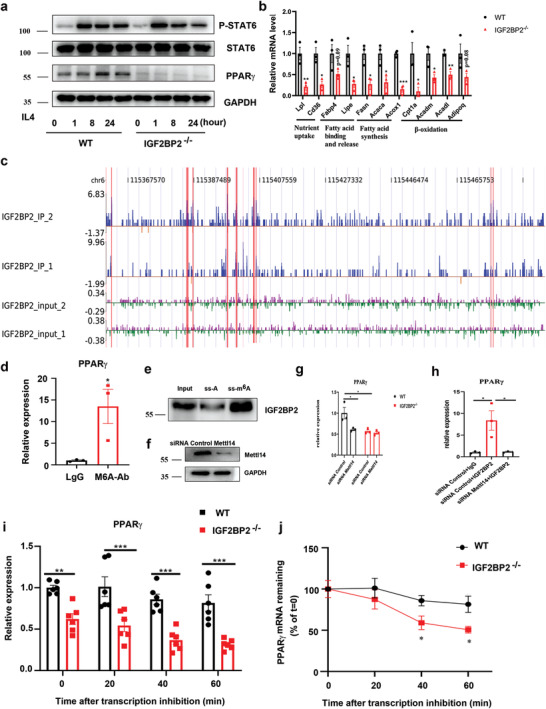
IGF2BP2 enhances PPAR*γ* mRNA stability via an m^6^A‐dependent manner. a) The immunoblot of STAT6, p‐STAT6, PPAR*γ*, and GAPDH in BMDMs after IL4 treatment at indicated time points; *n* = 5. b) Relative transcript levels of genes involved in fatty acid metabolism measured by RT‐qPCR after IL4 treatment BMDMs for 8 h; *n* = 3. c) The reads density landscape of IGF2BP2‐binding peaks on PPAR*γ* transcripts in BMDMs from IGF2BP2‐iRIP‐seq; *n* = 2. d) m^6^A enrichment of PPAR*γ* mRNA in BMDMs by m^6^A‐RIP‐qPCR. Results are presented relative to those obtained with immunoglobulin G (IgG); *n* = 3. e) Immunoblotting of IGF2BP2 in BMDMs after RNA pull down assay using single‐stranded PPAR*γ* RNA with methylated or unmethylated adenosine. f) The immunoblot of Mettl14 in WT BMDMs after siRNA transfection for 72 h; *n* = 3. g) WT or IGF2BP2^–/–^ BMDMs were interfered by siRNA Mettl14 for 72 h, the expression of PPAR*γ* was checked by RT‐qPCR; *n* = 3. h) RIP‐qPCR showing the enrichment of PPAR*γ* in BMDMs after siRNA Control and siRNA Mettl14 treatment; *n* = 3. i) RT‐qPCR of TSC1 mRNAs and j) TSC1 mRNA degradation in BMDMs treated with actinomycin D for the indicated times. The residual RNAs were normalized to 0 h; *n* = 6. Data were shown as mean ± SEM. *P < 0.05; **p < 0.01, ***p < 0.001 versus the WT group by b,d,h) two‐tailed unpaired Student's *t*‐test or g,i,j) two‐way ANOVA.

Given that PPAR*γ* developed a vital character in alternatively activated macrophages, which is facilitated by STAT6,^[^ ^26^ ^]^ and STAT6 induced expression of IGF2BP2 (Figure 2d), we further considered whether PPAR*γ* could be a target of IGF2BP2 to contribute to M2 activation. As expected, PPAR*γ* was almost vanished in IGF2BP2^–/–^ BMDMs compared with WT (**Figure** **3**a and Figure S6f, Supporting Information). PPAR*γ* is required for fatty acid metabolism genes expression, the tested genes including lipoprotein lipase (Lpl), fatty acid translocase (Cd36), fatty acid binding protein 4 (Fabp4), hormone sensitive lipase (Lipe), fatty acid synthase (Fasn), acetyl‐Coenzyme A carboxylase a (Acaca), acyl‐Coenzyme A oxidase 1(Acox1), carnitine palmitoyltransferase 1a (Cpt1a), medium‐ and long‐chain acyl‐CoA dehydrogenase (Acadm and Acadl) and adiponectin (Adipoq) were significantly reduced in IGF2BP2 null compared with WT BMDMs (Figure 3b). Additionally, iRIP also identified PPAR*γ* among the mRNAs bound by IGF2BP2 in two independent replicates (Figure 3c). To further explore the link between IGF2BP2 and PPAR*γ*, N6‐methyadeninosine (m^6^A) RNA‐real‐time polymerase chain reaction (qPCR) with M^6^A antibody, gene‐specific m^6^A pull down assay and RNA immunoprecipitation‐coupled qPCR (RIP‐qPCR) with IGF2PB2 antibody were used. We measured m^6^A levels of PPAR*γ* by MeRIP‐qPCR in BMDMs showing that m^6^A modified the mRNA of PPAR*γ* (Figure 3d). Through analysis of binding peaks of iRIP‐sequencing data, we designed methylated single‐stranded RNA bait (ss‐m^6^A) or unmethylated control RNA (ss‐A) for RNA pull‐down. Consistent with MeRIP‐qPCR, RNA pull‐down assay confirmed that IGF2BP2 protein selectively bind to the methylated bait than the unmethylated control (ss‐A) of PPAR*γ* (Figure 3e). METTL14 is one of “writers” in the process of m^6^A, and the METTL3‐METTL14 complex mediates mammalian nuclear RNA N6‐adenosine methylation. Our data showed that the relative level of PPAR*γ* with WT, but not those with null IGF2BP2, was decreased by Mettl14 silence (Figure 3f,g). Noticeably, we found the binding activity between IGF2BP2 and PPAR*γ* mRNA fragment was significantly reduced after knockdown of Mettl14 by siRNA (Figure 3h). IGF2BP2 can enhance mRNA stability and translation which are recognized by RNA N6‐methyladenosine.^[^ ^27^ ^]^ RNA decay assessment reported that PPAR*γ* mRNA was faster decay in IGF2BP2‐deficient than WT BMDMs (Figure 3i,j), suggesting that IGF2BP2 can increase PPAR*γ* mRNA stability. Taken together, these data demonstrated that the M2 activation deficiency of IGF2BP2^–/–^ macrophage was, at least in part, regulated by the STAT6‐IGF2BP2‐PPAR*γ* axis.

### 2.5 IGF2BP2 Regulates Both of M1 and M2 Activation by Targeting TSC1 in an m^6^A‐Dependent Manner

**Figure 4:**
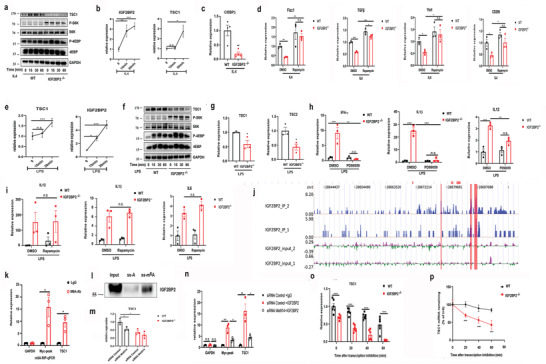
IGF2BP2 regulates macrophage activation via m^6^A modified TSC1/mTORC1 dependent and independent manner. a,f) Levels of TSC1, S6K, p‐S6K, 4EBP, and P‐4EBP in a) IL4‐stimulated or f) LPS‐stimulated BMDMs were determined by western blots; *n* = 5. b,e) BMDMs were stimulated with b) either IL4 or e) LPS at indicated time points. The TSC1 and IGF2BP2 mRNA expression were determined by real‐time PCR; *n* = 3. c) C/EBP*β* mRNA expression in BMDMs after IL4 treatment for 24 h; *n* = 4. d) BMDMs were pretreated with Rapamycin (40 nmol) for 30 min and then stimulated with IL4 for 24 h. TGF‐*β*, Fizz1, Ym1, and CD206 mRNA expressions were determined by RT‐qPCR; *n* = 3. g) TSC1 and TSC2 mRNA expression after LPS treatment BMDMs for 24 h; *n* = 3. h,i) BMDMs were pretreated with h) PD98059 (20 µmol) and i) Rapamycin (40 nmol) for 30 min and then costimulated with LPS for 24 h. IL1*β*, IFN‐*γ*, IL‐6, and IL12 expression were determined by RT‐qPCR; *n* = 3. j) The binding profiles of IGF2BP2 on TSC1 transcripts in BMDMs are shown; *n* = 2. k) m^6^A enrichment of TSC1 mRNA in BMDMs by m^6^A‐RIP‐qPCR. Results are presented relative to those obtained with immunoglobulin G (IgG). Glyceraldehyde 3‐phosphate dehydrogenase (Gapdh), m^6^A negative control; Myc peak, m^6^A positive control; *n* = 3. l) Immunoblotting of IGF2BP2 in BMDMs after RNA pull down assay using single‐stranded TSC1 RNA with methylated or unmethylated adenosine. m) Relative TSC1mRNA level in WT or IGF2BP2^–/–^ BMDMs with knockdown of Mettl14; *n* = 3. n) RIP‐qPCR showing the enrichment of TSC1 in BMDMs after siRNA Control and siRNA Mettl14 treatment; *n* = 3. o,p) RT‐qPCR of o) TSC1 mRNAs and p) TSC1 mRNA degradation in BMDMs treated with actinomycin D for the indicated times. The residual RNAs were normalized to 0 h; *n* = 6. Data were shown as mean ± SEM. n.s.: no significant, *P < 0.05; **p < 0.01, ***p < 0.001 versus the WT group by b,c,e,g,k) two‐tailed unpaired Student's *t*‐test or d,h,I,m,n,o,p) two‐way ANOVA.

IRS2/PI3K/Akt signaling is another activating signal downstream of IL‐4R, which has an invaluable potency in M2 polarization.^[^ ^28^ ^]^ Simultaneously, activated mTORC1 signaling blocks M2 polarization. Our findings suggested that mTORC1 was provoked in IGF2BP2 deficient BMDMs as indicated by enhanced phosphorylation level of the downstream targets S6K1 and 4E‐BP1 after IL4 treatment (**Figure** **4**a and Figure S6g, Supporting Information). Then we turn our sights to assess of expression of TSC1, which is a negative regulator of mTORC1 signaling and found TSC1 was downregulated in IGF2BP2^–/–^ BMDMs (Figure 4a and Figure S6g, Supporting Information). Additionally, TSC1 was upregulated following IGF2BP2 changes after IL4 stimulation (Figure 4b). One report shows that mTOR‐dependent nuclear C/EBP*β* play a dominant role in M2 macrophage polarization,^[^ ^29^ ^]^ which is similar to our study indicating that C/EBP*β* mRNA expression markedly decreased in IGF2BP2^–/–^ macrophages (Figure 4c). Importantly, by inhibiting mTORC1 activity after Rapamycin treatment, M2 related genes expression were partially reversed in IGF2BP2^–/–^ macrophages (Figure 4d).

Given that metabolic remodeling is associated with macrophage activation status, we compared WT with IGF2BP2^–/–^ macrophages aerobic respiration of M2 macrophages by using seahorse assays. Notably, upon IL4 activation, oxygen consumption rates (OCRs) (Figure S4a,b, Supporting Information) and spare respiratory capacity (SRC) (Figure S4c, Supporting Information), which represent oxidative phosphorylation (OXPHOS) activity, exhibit diminished in IGF2BP2^–/–^ BMDMs. Moreover, the OCRs and SRC in TSC1 knockdown BMDMs were consistent with IGF2BP2‐deficient BMDMs (Figure S4, Supporting Information).

Because TSC1 also regulates M1 polarization,^[^ ^29^ ^]^ to further explore potential mechanism how IGF2BP2 affects M1 macrophages differentiation, we assessed the mTORC1, TSC1 and IGF2BP2 activity in LPS stimulated BMDMs. Similarly, the mTORC1 signaling was enhanced in IGF2BP2^–/–^ BMDMs compared with WT BMDMs (Figure 4e,f and Figure S6h, Supporting Information). TSC1 can stabilize TSC2 and previous studies have been demonstrated that TSC1/2 complex regulated MEK/ERK activity to affect inflammatory M1 polarization.^[^ ^29^ ^]^ In line with their results, TSC2 mRNA level was also downregulated in IGF2BP2 deficient macrophages (Figure 4g) and treatment of the specific MEK1/2 inhibitor PD98059 significantly saved the elevated IL1*β*, IFN‐*γ* and TNF‐α production in IGF2BP2‐deficient macrophages after LPS stimulation (Figure 4h). However, rapamycin treatment failed to rescue the enhanced IL‐1*β* and IL12 expression (Figure 4i), which corresponds with the published report of TSC1 deficiency macrophages.

Based on the properties of IGF2BP2, as an mRNA binding protein, we finally sought to investigate whether IGF2PB2 interacts with TSC1 directly. The iRIP‐Seq also confirmed that IGF2BP2 directly targeted the TSC1 transcripts in macrophages (Figure 4j). MeRIP assays, M^6^A‐RNA pull down and RIP assays was then performed in BMDMs. Notably, TSC1 engaged in IGF2BP2 dependent m^6^A modification (Figure 4k,m). Similar to PPAR*γ*, IGF2BP2 proteins also preferentially bound to methylated RNA of TSC1 over the unmethylated one (Figure 4l) and the TSC1mRNA levels upon siRNA Mettl14 depends on the presence of IGF2BP2 (Figure 4m). Consistently, a strong binding of TSC1 with IGF2BP2 in WT BMDMs and a much less or no binding in siRNA Mettl14 WT BMDMs (Figure 4n). To explore whether IGF2BP2 also had impact on the stability of TSC1 mRNA, we treated BMDMs with actinomycin D in indicated time points before total RNA was acquired. The half‐life of TSC1 was notably shortened, roughly 50%, in IGF2BP2 deficient BMDMs (Figure 4o,p). These findings collectively supported that IGF2BP2 regulated M2/M1 polarization via TSC1/mTORC1 and TSC1/2/MEK/ERK pathway respectively and IGF2BP2 served as an m^6^A reader to increase TSC1 stability in the processing of methylation.

### 2.6 IGF2BP2 Expression in Hematopoietic Cells Is Involved in Preventing the Development of Colon Inflammation

**Figure 5:**
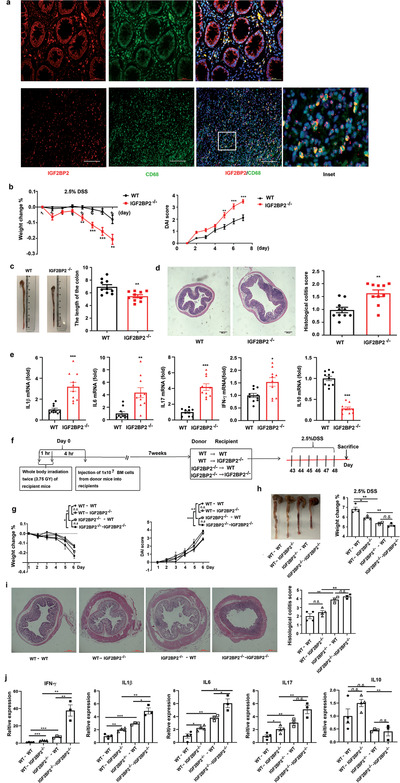
IGF2BP2^–/–^ immune cells predispose mice to colitis. a) IGF2BP2 (red) is coexpressed with CD68 (green) in individual cells and cellular nuclei were labeled by 4’,6‐diamidino‐2‐phenylindole (DAPI, blue); *n* = 3 (biological repeats) human normal (up) or colonic tissues (down). Scale bars: 50 µm. b) Body weight change was shown as a percentage of initial body weight and the disease activity index (DAI) was scored; *n* = 10. c) The colon length was measured after the mice were sacrificed; *n* = 10. d) Histopathological changes and semi‐quantitative scoring of histopathology in colon tissue from wild‐type and IGF2BP2^–/–^ mice were analyzed by hematoxylin and eosin (H&E) staining on day 7 of DSS administration. Scale bars, 200 µm; *n* = 10. e) Cytokine production in whole cells from colonic LP of wild‐type and IGF2BP2^–/–^ mice after 7 d of DSS administration; *n* = 10. f–j) Contribution of immune and nonimmune cells derived IGF2BP2 on the severity of colitis in IGF2BP2 bone marrow chimeric (*n* = 4 for WT→WT, WT→IGF2BP2^–/–^, *n* = 3 for IGF2BP2^–/–^→WT, IGF2BP2^–/–^→IGF2BP2^–/–^). f) Protocol for the generation of bone marrow chimeras. CD45.2^+^WT and CD45.2^+^ IGF2BP2^–/–^ recipient mice were lethally irradiated with 7.5 Gy (two divided doses 3 h apart) and injected intravenously (i.v.) with 1 × 10^7^ BM cells per mouse from CD45.1^+^WT or CD45.1^+^IGF2BP2^–/–^ mice. The following four chimera groups were generated: WT→WT, WT→IGF2BP2^–/–^, IGF2BP2^–/–^→WT, IGF2BP2^–/–^→IGF2BP2^–/–^ (referred as “donor into recipient mice”). g) Body weight changes and DAI were monitored for 6 d. h) Representative colon photographs and the average colon lengths of mice after 6 d of DSS treatment are shown. i) Histological Hematoxylin and eosin (HE) staining of colon‐paraffin sections and the histological HE staining score of colon‐paraffin sections in mice. Scale bars, 200 µm. j) Cytokine mRNA expressions in colon tissues were measured by real‐time PCR after 6 d of DSS treatment. Data were shown as mean ± SEM. *P < 0.05, **P < 0.01, ***P < 0.001. *P*‐values were determined by using c,d,e) unpaired, two‐tailed Student's *t*‐test or b,g,h,i,j) two‐way ANOVA. Data are representative of two independent experimental replicates and are presented as means ± SEM.

Next, we assessed whether IGF2BP2 involved in the regulation of inflammatory diseases in vivo. Inflammatory macrophages are implicated in the pathogenesis of ulcerative colitis development.^[^ ^30^ ^]^ Consistent with higher levels of IGF2BP2 in LPS treated macrophages (Figure 1 and Figure S1, Supporting Information), we found that IGF2BP2 were positive in approximately half of CD68^+^ macrophages from ulcerative colitis patients (**Figure** **5**a). Then, we evaluated the impact of IGF2BP2 in dextran sulfate sodium (DSS)‐induced colitis mice model. After 7 d 2.5% DSS administration, IGF2BP2^–/–^ mice displayed more severe colitis compare with WT mice, associated with faster rate of body weight loss, higher disease activity index (DAI) (Figure 5b) and tinier colon length (Figure 5c). Moreover, the degree of infiltration of inflammatory cells and crypt destruction were more severe in IGF2BP2^–/–^ than WT mice analyzed by H&E staining (Figure 5d). Similarly, lamina propria (LP) cells from DSS‐treated IGF2BP2^–/–^ mice displayed severely impaired IL‐10 secretion, and M1 macrophages markers were markedly elevated (Figure 5e). We have shown that IGF2BP2 deficiency rendered mice prone to the acute inflammation. To define whether the development of colon inflammation in IGF2BP2^–/–^ mice was due to its absence in the hematopoietic or nonhematopoietic cell compartment, we generated bone marrow chimera mice by crisscross transplantation of WT or IGF2BP2^–/–^ bone marrow cells (BMCs) to WT or IGF2BP2^–/–‐^ mice (see protocol in Figure 5f). Full chimera mice (WT → WT, WT → IGF2BP2^–/–^, IGF2BP2^–/–^ → WT, IGF2BP2^–/–^ → IGF2BP2^–/–^) were confirmed by flow cytometry assays (data not shown) and then subjected to DSS‐induced epithelial damage in 7 weeks after bone marrow transplantation. Mice with hematopoietic IGF2BP2 deficiency (IGF2BP2^–/–^ → WT, IGF2BP2^–/–^ → IGF2BP2^–/–^) had more severe disease relative to mice with IGF2BP2 expression in hematopoietic cells (WT → WT, WT → IGF2BP2^–/–^), as observed by substantial body weight loss (Figure 5g), increased DAI (Figure 5g), shortened colon length (Figure 5h), severe colon damage (Figure 5i), significant histological inflammatory cell infiltration (Figure 5i) and elevated production of proinflammatory cytokines (Figure 5j) in colons. Interestingly, in IGF2BP2 knockout mice receiving WT BMCs (WT → IGF2BP2^–/–^) the production of proinflammatory cytokines was more severe relative to WT mice receiving WT BMCs (WT → WT) (Figure 5g–j), indicating that knockout IGF2BP2 also affects the release of inflammatory cytokines in other cells. These data suggested IGF2BP2 deficiency macrophages exacerbated DSS induced colitis in mice.

### 2.7 IGF2BP2 Skews toward M2 Polarized in a Chitin Challenged in Vivo Model

We also tried to corroborate our in vitro results of the IGF2BP2 effect on M2 macrophages activation. The function of IGF2BP2 in PMs were investigated by using a murine chitin administration model. Chitin mediated IL‐4‐dependent M2 alternative activation in vivo, which was required for optimal immune cell recruitment to the site of chitin delivery.^[^ ^31^ ^]^ We evaluated the M2 macrophages markers after injection of chitin and observed a profound reduction of M2 gene expression in IGF2BP2^–/–^ peritoneal exudate cells (Figure S5, Supporting Information). Given that, our findings supported that IGF2BP2 was critically involved in acquisition of anti‐inflammatory properties in vivo.

### 2.8 IGF2BP2^–/–^ Hematopoietic Cells Alleviate Cockroach Allergen Induced Lung Inflammation

**Figure 6:**
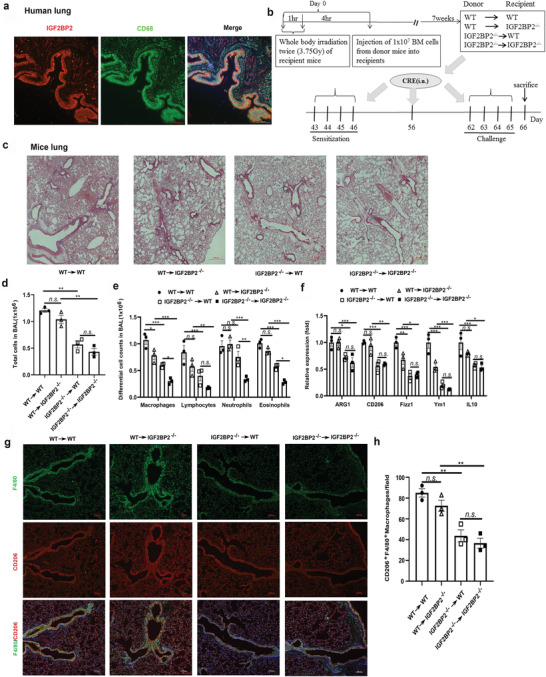
IGF2BP2 deficient myeloid cells are able to alleviate CRE‐induced asthma. a) IGF2BP2 (red) is coexpressed with CD68 (green) in asthmatic cells and cellular nuclei were labeled by DAPI (blue); *n* = 3 (biological repeats). Scale bars: 100 µm. b) Protocol for cockroach allergen‐induced bone marrow (BM) chimeric mouse model of asthma including three mice per group. c) Paraffin‐embedded tissue sections of lungs from CRE‐challenged recipient mice were stained with H&E, Scale bars: 100 µm. d) Total BAL fluid cells and e) differential BALF cells numbers in CRE‐challenged recipient mice. f) The levels of M2‐associated genes ARG1, CD206, Fizz1, Ym1, and IL10 in lung tissue samples were quantified by RT‐qPCR. g) CD206 (red) is coexpressed with F4/80 (green) in individual cells and cellular nuclei were labeled by DAPI (blue) in CRE‐challenged lung tissues. Scale bars: 100 µm. h) Quantification of F4/80, CD206 positive cells of lung tissues after CRE‐challenged. Data are presented as means ± SEM. *P < 0.05; **P < 0.01; ***P < 0.001. *P*‐values were determined by using unpaired two‐way ANOVA.

We have shown that IGF2BP2 rendered macrophages prone to M2 polarization (Figures 1, 2, 3, 4). Moreover, the expression of IGF2BP2 was detected in asthmatic and it was mostly colocalized with macrophages in human asthma samples (**Figure** **6**a). To further assess whether IGF2BP2 in macrophages plays a role in M2 polarization in vivo, we also used cockroach allergen (CRE) to induce allergic lung inflammation in bone marrow chimera mice (Figure 6b), in which M2 macrophages played a critical role as shown before.^[^ ^32^ ^]^ Wild‐type (WT → WT) chimeras had more severe asthma pathologies than IGF2BP2‐deficient chimeric mice (IGF2BP2^–/–^ →IGF2BP2^–/–^). With respect to IGF2BP2 deficiency in hematopoietic cells, following CRE treatment, IGF2BP2^–/–^ mice reconstituted with WT bone marrow cells (WT →IGF2BP2^–/–^) exhibited asthma signs as severe as those in wild‐type mice (WT → WT). Conversely, mice lacking hematopoietic IGF2BP2 (IGF2BP2^–/–^ → WT) exhibited asthma pathologies comparable to wild type mice (WT → WT), concomitant with significantly recruitment of inflammatory cells to the lungs, dense peribronchial infiltrates (Figure 6c), the total number in the bronchoalveolar lavage fluid (BALF) (Figure 6d), as well as macrophages, lymphocytes, neutrophils, and eosinophils among all analyzed cell types in BALFs (Figure 6e). Subsequently, we examined the levels of M2‐associated gene levels, as expected, levels of ARG1, CD206, Ym1, Fizz1, and IL10 were much lower in lung tissues from IGF2BP2^–/–^ →WT mice than WT → WT mice (Figure 6f). Moreover, compared with CRE‐treated WT → WT mice, decreased levels of M2 macrophages were observed in lung tissues from CRE‐treated IGF2BP2^–/–^ → WT mice (Figure 6g,h). Together, these findings implied that IGF2BP2 in hematopoietic cells might tip the balance of macrophages toward the M2 subset during CRE‐induced allergic inflammation, thereby exacerbating pulmonary inflammation development.

## 3 Discussion

IGF2BPs are increasingly implicated in modulating varies of biological processes, including development, tumorigenesis, and stemness. A recent finding shows that IGF2BP2 is required for their recognition of m^6^A modifications and is critical for mRNA stability and translation.^[^ ^27^ ^]^ However, it remains unclear whether IGF2BP2 involves in macrophage polarization as an m^6^A reader. In the present study, we showed that the expression of IGF2BP2 increased obviously after macrophage differentiated to both of M1 and M2 and this IL‐4 induced upregulation of IGF2BP2 was in an STAT6 dependent manner. Using loss‐of‐function approach, we demonstrated that IGF2BP2 acted as a positive regulator of IL‐4 driven M2 activation but negatively modulated proinflammatory response. Further m^6^A‐RIP‐qPCR analysis identified that TSC1 is an m^6^A modified target recognized by IGF2BP2. TSC1 is well known to be a critical regulator that controls macrophage polarization. Mice with myeloid‐specific deletion of TSC1 spontaneously develop M1‐related inflammatory disorders. However, TSC1‐deficient mice are highly resistant to M2‐polarized allergic asthma.^[^ ^29^ ^]^ This suggests that IGF2BP2 exerts its function on macrophage activation partially via TSC1, adding a new dimension to the essential function of m^6^A modification in the physiology of macrophages.

Role of m^6^A modifications in the immune system has been demonstrated in controlling varies aspects of immunity, including immune recognition, activation of innate and adaptive immune responses, and immune cells fate determination. For instance, specific depletion of METTL3 in CD4^+^ cells impaired T cell homeostasis and differentiation.^[^ ^33^ ^]^ In innate host cells, the m^6^A eraser ALKBH5 was decreased once viral infection, further rewiring the cellular metabolism to inhibit viral replication.^[^ ^34^ ^]^ FTO is another important eraser in RNA methylation machinery, FTO depletion impeded M1 activation thro reducing ugh mRNA stability of STAT1 via the m^6^A reader YTHDF2 mediation respectively.^[^ ^35^ ^]^ In addition, another independent group reported that YTHDF2 knockdown significantly produced more proinflammatory cytokines by increasing expression and stability of MAP2K4 and MAP3K4.^[^ ^36^ ^]^ Paradoxically, knockdown of the m^6^A writer METTL3 also inhibited M1 BMDM polarization and downregulated STAT1 expression.^[^ ^37^ ^]^ Here, knockout of the m^6^A reader IGF2BP2 rendered macrophages highly susceptible to M1 polarization, whereas we did not see any obvious change of STAT1 expression in M1 IGF2BP2^–/–^ BMDMs (Figure S1e, Supporting Information). Instead, our data showed that the IGF2BP2‐TSC1/2‐ERK axis was responsible for negative regulation of macrophages activation in response to LPS stimulation. More studies will be needed to decipher these divergent roles of m^6^A modification on M1 macrophage activation.

Recently, although the RNA‐binding proteins have emerged as regulators of development and function of immune cells, their roles in the inflammatory diseases are only beginning to be explored.^[^ ^38^ ^]^ Our results provide a novel evidence that the RNA‐binding protein IGF2BP2 contributes to the inflammation development in the colon and lung by controlling macrophages polarization. In the DSS induced experimental acute colitis mice model, the IGF2BP2 deficient mice exhibited an enhanced colitis phenotype characterized by more body weight loss, shorter colon length, more severe epithelial damage, and higher inflammatory cytokines production. Upon intestinal epithelial cells damage, the epithelial cells and dendritic cells detects damage‐associated molecular patterns and pathogen‐associated molecular patterns and attracts circulating neutrophils. Infiltrating neutrophils in turn recruit LY6C^int^CX3CR1^int^ inflammatory monocytes to mount an appropriate response to the inflammogen, these LY6C^int^CX3CR1^int^ cells retain their ability of secreting inflammatory cytokines including IL‐12, IL‐23, and IL‐1*β*, by that trigger Th1 and Th17 immune responses and aggravate tissue damage. Growing evidence reveals that imposing the inflammatory macrophages into a resolving phenotype is benefit to control colonic inflammation and accelerate tissue repair.^[^ ^30^ ^]^ In accordance with our in vitro results, we observed that more nitric oxide synthases (iNOS)^+^ macrophages infiltrated into the colonic epithelial cells in the IGF2BP2 null mice. Conversely, IGF2BP2 deficiency ameliorated CRE induced allergic asthma, and knockout of IGF2BP2 constrained M2 macrophages polarization. Indeed, clinical data show that alternative macrophages are associated with asthma induction and progression.^[^ ^39^ ^]^ Thus, our findings provide a potential target to modulate macrophages activation for treating intestinal and pulmonary inflammation. Additionally, the iRIP‐seq identified a large amount of potential IGF2BP2 targets that could involve in varies of inflammatory diseases (Figure S3a, Supporting Information), for example, Ifi204 could control the IRF7‐mediated type I interferon response negatively during RNA virus infection to avoid hyper‐inflammatory responses.^[^ ^40^ ^]^ Thus, further investigation to explore whether IGF2BP2 involves in other inflammatory diseases can be helpful to fully understand the role of IGF2BP2 in human diseases.

The high plasticity of macrophages relies on metabolic reprogramming, in particular, M2 macrophages are mainly dependent on OXPHOS and FAO. Multiple studies have highlighted that IGF2BP2 plays a significant role in metabolism of type 2 diabetes and cancer. For example, IGF2BP2 inhibits UCP1 translation and series of mitochondrial polypeptides in brown adipose tissue.^[^ ^41^ ^]^ Moreover, IGF2BP2 modulates mitochondrial activity and encodes mitochondrial respiratory chain complex subunits in glioblastoma cancer stem cells.^[^ ^42^ ^]^ Our finding that loss of IGF2BP2 impaired OXPHOS in IL4 induced M2 macrophages provides more evidence for these early findings. Recent report showed that IGF2BP2 stabilizes HK2 and GLUT1 mRNA to promote glycolysis in colon cancer ^[^ ^43^ ^]^ and glycolysis is necessary for M2 macrophage activation.^[^ ^25^ ^]^ These researches show another possibility that IGF2BP2^–/–^ macrophages have deficient functions of glucose uptake and glycolysis to support M2 polarization. Additionally, our data also indicate that IGF2BP2 can bind to PPAR*γ* directly in an m^6^A dependent manner and PPAR*γ* involves in FAO metabolism and controls macrophage alternative activation.^[^ ^44^ ^]^ Collectively, the IGF2BP2 protein master different genes expression via post‐transcriptional modification, which are indispensable for M2 macrophage metabolic reprogramming.

**Figure 7:**
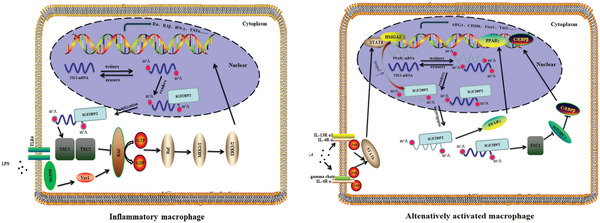
Schematic diagram of IGF2BP2‐mediated regulation of macrophages activation. Graphical view of IGF2BP2 effects on inflammatory macrophage (left) and alternatively activated macrophage (right).

In summary, our results highlight a key role of m^6^A modifications in control of macrophage polarization. Defect of IGF2BP2 promoted M1 response, leading to enhancing DSS induced experimental colitis development, but impaired M2 activation through stabilizing TSC1 and PPAR*γ* mRNA. Together, our new identification of the master role of IGF2BP2 in macrophages differentiation (**Figure** **7**) may help to further understand the pathophysiological function of m^6^A modifications in many macrophage‐directed sites, including inflammation, allergy, and tumor progression.

## 4 Experimental Section

##### Animals

IGF2BP2 deficient mice were generously provided by Dr. Liu at the National Research Center for Assisted Reproductive Technology and Reproductive Genetics, Shangdong University, China.^[^ ^45^ ^]^ IGF2BP2^–/−^ C57BL/6J background mice were generated by back crossing with the C57BL/6J wild type mice for at least six generations. Sex‐ and age‐matched littermate mice were used in all studies. All mice were bred and maintained in specific pathogen‐free conditions in the animal facility of the School of Basic Medical Sciences. All animal experiments were conducted (#ECSBMSSDU2019‐2‐048) in accordance with the guidelines of the Animal Care and Use Committee of Shandong University.

##### DSS‐Induced Colitis Model

2.5% DSS salt (reagent‐grade, mol. wt. 36 to 50 kDa; MP Biomedicals) was used which was dissolved in autoclaved drinking water to induced colitis. Mice were sacrificed and calculated the severity of colitis by DAI containing the combined score of bleeding, stool consistency, and body weight from day 0 to 7. Briefly, it is the average score of body weight change (scored as: 0, none; 1, 1–5%; 2, 5–10%; 3, 10–15%; 4, > 15%), stool consistency (scored as: 0, normal; 1 and 2, loose stool; 3 and 4, diarrhea), and bleeding (scored as: 0, negative; 1, +; 2, ++; 3, +++; 4, ++++).^[^ ^46^ ^]^ After 7 d DSS administration, colon was dissociated and measured the length from the ileocecal junction to the anal verge. After quickly and gently washed by precooled PBS, proximal rectal colon was cut into five 1 cm segments then planted in 4% paraformaldehyde and the distal parts were kept in RNA fixer (Aidlab) for isolating RNA later.

##### Patient Samples

The colonic and asthma tissue biopsies used for this study were obtained from Qilu Hospital of Shandong University and informed consent was provided from all patients. Patients were diagnosed with ulcerative colitis. From the resected colon segment, normal as well as colitis tissue was isolated. The use of human tissues was approved (#LL‐201601005) by the Medical Institutional Ethics Committee of Qilu Hospital, Shandong University, China.

##### Cockroach Allergen‐Induced Mouse Asthma Model

Generation of a cockroach allergen‐induced mouse model of asthma and analysis of lung inflammation was established as described previously.^[^ ^47^ ^]^ Female mice were sensitized with intranasal delivery of cockroach extract (CRE; B46, Greer Laboratories) at a concentration of 20 µg per mouse on day 0, 1, 2, 3, and 14 and challenged with the same amount of CRE for four successive days (day 20–23). Control mice received the same volume of PBS. On day 24, mice were sacrificed, BALF and lungs were harvested for the proposed studies.

##### Analysis of Lung Inflammation

Detailed methods were described previously.^[^ ^48^ ^]^ Mouse lungs were fixed in 4% formaldehyde overnight and embedded in paraffin. Five‐micrometer lung sections were cut and then stained with H&E and immunofluorescence to evaluate general morphology. Right lungs were used to extract RNA and alveolar macrophages. For analysis of BALF, mouse lungs were per‐fused with 1 mL ice‐cold PBS three times, and lavage fluids were collected and centrifuged at 1500 rpm at 4 ˚C for 10 min. After red blood cells (RBCs) were lysed, total cell number was counted by using a hemocytometer (Hausser Scientific), and cellular differential percentage was determined by flow cytometry.

##### Isolation and Culture of Cells

Mouse BMDMs were isolated and cultured as previously described^[^ ^49^ ^]^ Briefly, bone marrow was extracted from femur and tibia, after lysis of erythrocytes, marrow cells were cultured for 7 d in complete medium consisting of Dulbecco's modified Eagle's medium with 30% L929‐conditioned medium and 10% heat‐inactivated fetal bovine serum. For isolation of mouse peritoneal macrophages, peritoneal cavity exudate cells were obtained by washing of the cavity with lavage media comprised of dulbecco's modified eagle medium (DMEM) containing 200 U mL^−1^ penicillin and 100 µg mL^−1^ streptomycin. Mouse lung macrophages was performed according to published protocols.^[^ ^32^ ^]^ Lung tissues were minced and incubated for 45 min at 37 °C in Dulbecco modified Eagle medium supplemented with 10 µg mL^−1^ DNAse I and 1 mg mL^−1^ collagenase D (Sigma‐Aldrich, St Louis, Mo) in a shaking water bath. Digested lung tissues were then passed through a 70 µm nylon strainer (BD Biosciences, San Jose, CA) to obtain a single‐cell suspension. For M1‐like activation, (0.5–0.7)×10^6^ macrophages were plated in tissue culture dishes and treated with 100 ng mL^−1^ LPS (Invivogen). For M2 polarization, cells were treated with 20 ng mL^−1^ IL‐4 (Peprotech). BMDMs were cultured 7 d in complete medium and stimulated in 12‐well culture plates. Cells were pretreated with the inhibitors Rapamycin, Ruxolitinib, AG‐490, PP242, PD98059, JANEX‐1, Wortmannin, and AS1517499 for 30 min and then stimulated with IL4 or LPS and inhibition in the indicated time and concentrations. All reagents were obtained from Selleck.

##### Histology and Immunofluorescence Microscopy

For histopathological analysis, colon and lung samples (5 µm) were stained with hematoxylin and eosin (H&E) after dehydration embedding. The histological analysis was performed as previously described,^[^ ^50^ ^]^ including the degree of inflammation (scored as: 0, none; 1, Slight; 2, Moderate; 3, Severe), the area of intestine layers (scored as: 0, none; 1, mucosa; 2, submucosa; 3, transmural), the extent of ulceration (scored as: 0, none; 1, mild ulceration; 2, moderate ulceration; 3, extensive ulceration), the damage of crypt (scored as: 0, none; 1, submucosa; 2, basal one‐third lost; 3, basal two‐thirds lost; 4, only surface epithelium intact; 5, all crypts and epithelium are destroyed) and the percentage of involvement (scored as: 0, none; 1, 1–25%; 2, 26–50%; 3, 51–75%; 4, 76–100%). For immunofluorescence, after antigen retrieval performed by sodium citrate buffer (Solarbio) in Pansonic for 30 min and blocked with donkey serum (Solarbio) for 45 min to avoid nonspecific staining, Slides were incubated with primary antibodies such as IGF2BP2, CD68, F4/80, INOS, and CD206 at 4 °C overnight. In the second day, secondary antibody with different excitation light such as Alexa Fluor 568 donkey antirabbit and Alexa Fluor 488 donkey antigoat (life technologies) was adopted for 1 h at 37 °C. Then DAPI (Beyotime) was used to counterstained nucleus. The antibody used in this study is written in Table S2 in the Supporting Information. The immunofluorescence staining analyze were performed double blindly by using image‐pro plus software.

##### RNA Extraction and Real‐Time PCR Analysis

The level of mRNA expressions was defined by reverse transcription polymerase chain reaction and RT‐qPCR. RNA was gained from tissue or cells with EASYspin Plus kit (Aidlab) and then synthesized to QuantiTect RevComplementary DNA (cDNA) by using the QuantiTect Rev. Transcription Kit (Vazyme, Nanjing) and augmented by using SYBR Green qPCR Mix (Vazyme, Nanjing) on Bioer‐ Lightcycler. △△Ct values were normalized to GAPDH, and relative quantification of gene expression was compared to WT group. The primers used in this study are noted in Table S1 in the Supporting Information and synthesized by the Beijing Genomics Institute (Beijing, China).

##### Western Blotting Analysis

Macrophages were washed three times in cold PBS, lysed in radioimmunoprecipitation assay (RIPA) buffer (Beyotime) with protease and phosphatase inhibitor cocktails (selleck) for 10 min on a rocker 100 rpm at 4 °C. Protein concentrations were quantified by using a bicinchoninic acid (BCA) protein kit (Beyotime). Proteins samples were analyzed on sodium dodecyl sulfate (SDS) polyacrylamide gel electrophoresis and transferred onto polyvinylidene fluoride or polyvinylidene difluoride (PVDF) membranes (Millipore). These membranes were blocked with 5% bovine serum albumin (BSA) (Solarbio) and 0.1% Tween 20 in Tris‐buffered saline 1 h, and then incubated overnight at 4 °C with primary antibody. The appropriate horseradish peroxidase (HRP)‐coupled secondary antibody was then added and was detected with chemiluminescent substrate BrightTM enhanced chemiluminescent (ECL) (Beyotime). The antibody used in this study is listed in Table S2 in the Supporting Information.

##### Chitin Administration

Chitin (Sigma) was washed three times with PBS and large aggregates settled for 2 min, suspended chitin adjusted to a concentration of 4 µg mL^−1^ and sonicated for 30–40 min on ice, filtered. A dose of 800 ng of chitin was injected intraperitoneal injection (i.p.) to induce recruitment and polarization of M2 cells into the peritoneum, and peritoneal exudate cells (PECs) were collected by lavage after 48 h and gene induction was determined by quantitative PCR.^[^ ^31^ ^]^

##### Flow Cytometry

For surface staining cells were stained with Zombie NIR Fixable Viability Kit (BioLegend), blocked with 5 µg mL^−1^ anti CD16/32 (BioLegend), and stained with fluorescently labeled antimouse antibodies diluted in PBS at an indicated concentration for 30 min at 4 °C. Intracellular staining was performed after incubation in fixation and permeabilization buffer (eBioscience Foxp3/Transcription Factor Staining Buffer Set). Cells were then immuno‐stained following the same protocol described above. After washing, cells were acquired by using Gallios (Bechman) and data were analyzed by using FlowJo software (Version 10; Tree Star). Eosinophils were defined as side scatter (SSC) high SiglecF^+^ cells, macrophages were identified as SSC high CD11b^+^ F4/80^+^ cells or CD11b^+^ F4/80^+^ PD‐L2^+^RELMα^+^ cells, M2 macrophages were recognized as SSC ^high^ Ly6G^–^ SiglecF^–^ CD3^–^ CD19^–^ CD11b^+^ F4/80^+^ CD206^+^ cells, granulocytes were recognized as SSC ^high^ Ly6G^+^ cells, and lymphocytes were identified as forward scatter (FSC)^low^/SSC^low^ cells expressing CD3 or CD19. The samples were incubated with antibodies as described in Table S2 in the Supporting Information.

##### Oxygen Consumption and Extracellular Acidification Rate Analysis

OCR and extracellular flux acidification rate were measured in BMDMS (80 000 cells per well density) with the XFe‐96 Extracellular Flux Analyzer. Cells were pre‐equilibrated for 1 h in unbuffered XF assay medium supplemented with 25 mmol glucose, 1 mmol sodium pyruvate, and 2 mmol l‐glutamine. For mitochondrial fitness tests, three or more consecutive measurements were taken under basal conditions and following the sequential addition 1 µmol oligomycin to inhibit the mitochondrial adenosine triphosphate (ATP) synthase, 1.5 µmol fluoro‐carbonyl cyanide phenylhydrazone a protonophore, which uncouples ATP synthesis from oxygen consumption by the electron transport chain, and 100 nmol rotenone plus 1 µmol antimycin A (Rot/Ant) to inhibit the electron transport chain (all purchased from Agilent Technologies). XFe Wave software (Seahorse Bioscience) was used to analyze the results and BMDMs were stimulated for 8 h with IL‐4 before analysis.

##### RNA Immunoprecipitation (RIP) Assays

RNA immunoprecipitation was performed as previously described with some modifications.^[^ ^27^ ^]^ RIP was conducted with the Magna RIP RNA‐Binding Protein Immunoprecipitation Kit (Millipore) according to the manufacturer's instructions. Briefly, 1 × 10^7^ cells were lysed by complete RIP Lysis Buffer. Magnetic beads coated with 5 µg of specific antibodies against mouse immunoglobulin G (Millipore), or IGF2BP2 (bethyl) were incubated with prepared cell lysates overnight at 4 °C. After washing RNA protein complexes with RIP buffer for 6 times, all tubes were incubated in proteinase K buffer at 55 °C for 30 min to digest the protein. RNA was finally extracted by phenol‐chloroform RNA extraction methods. Following centrifugation at 14 000 rpm for 15 min at 4 °C, the supernatant was removed carefully and the pellets were dried in the air, then resuspended in 15 µL of RNase‐free water. The relative interaction between IGF2BP2 and TSC1 transcripts was determined by qPCR and normalized to the input.

##### MeRIP‐qPCR

For m^6^A RIP, m^6^A antibody was used to pull down m^6^A modified individual genes. Briefly, total RNA was extracted by EASYspin Plus kit (Aidlab) and dissolved in 40 µL RNase‐free water, of which 2 µL was kept as RNA input, then the remaining RNA volume was adjusted to 1 mL buffer containing RNase inhibitor, ribonucleoside vanadyl complexes, m^6^A‐specific antibody (Sigma‐Aldrich) or rabbit IgG (Sigma‐Aldrich), subsequently was rotated for 2 h at 4 °C, then the prewashed beads A were added into the samples and reincubated for another 2 h. Next, the elution buffer containing anti‐m^6^A antibody (Synaptic Systems) was added to the mixture and incubated for 1 h with continuous shaking at 4 °C. The methylated mRNAs were precipitated with 5 mg of glycogen and one‐tenth volumes of 3 mol sodium acetate in a 2.5 volume of 100% ethanol at − 80 °C overnight. The m^6^A bound RNA was calculated by qPCR and the corresponding m^6^A enrichment was calculated by normalizing to the input.

##### RNA Stability Assay

The turnover rate or stability of mRNA in vivo is usually reported as the time required for degrading 50% of the existing mRNA molecules.^[^ ^51^ ^]^ To detect objective RNA stability, BMDMs were seeded in 12‐well plates and treated with 5 µg mL^−1^ actinomycin D (MedChemExpress) and then collected at the indicated time points. Total RNA was extracted by EASYspin Plus kit (Aidlab) and analyzed by RT‐PCR. The mRNA half‐live time were estimated according to the linear regression analysis.

##### Transfection of siRNA

Cells were transfected with Mettl14 siRNAs, TSC1siRNAs, and Control siRNAs by using INTERFERin (Polplus) according to the manufacturer's instruction. BMDMs were transfected with 50 nmol siRNA and 10 µL of transfection reagent on three consecutive days and experiments were performed 72 h after first transfection. The targeting siRNA sequences were designed and synthesized by Genepharma Company (Shanghai) and they are listed in Table S1 in the Supporting Information.

##### ChIP Assays

ChIP was performed using the SimpleChIP Plus Enzymatic Chromatin IP Kit cell signaling technology (CST). BMDMs Cells (5 × 10^6^) were fixed with 1% formaldehyde for 10 min at RT and the crosslinking was quenched by add glycine. Crosslinked chromatin was digested with 0.35 µL Micrococcal Nuclease and sonicated with three sets of 20 s pulses. For Chromatin Immunoprecipitation, samples were incubated with 10 µL ChIP‐grade anti‐STAT6 (CST) antibody and 30 µL ChIP‐Grade Protein G Magnetic Beads at 4 °C with rotation, after analysis of chromatin digestion and concentration. Normal rabbit IgG was used as a negative control. Following Immunoprecipitation, DNA was reverse crosslinking and purified according to the manufacturer's instructions and analyzed by RT‐qPCR. ChIP results were calculated as percentage of input DNA. The primers and antibody for ChIP‐assays are listed in Tables S1 and S2 in the Supporting Information. Putative STAT6‐binding sites were predicted by JASPAR database (http://jaspar.genereg.net/).

##### Bone Marrow Chimeras

IGF2BP2 deficient chimeric mice were generated by total body irradiation followed by bone marrow transfer.^[^ ^52^ ^]^ Briefly, 6–8‐week‐old WT and IGF2BP2^−/−^ recipient mice (expressing CD45.2 leukocyte antigen) were subjected to 7.5‐Gy (in two divided doses, 1 h apart) lethal irradiation with an X‐ray (PXi). After 4 h later, the recipient mice were injected intravenously with BM cells (1 × 10^7^) prepared from the femur and tibia of donor mice (expressing CD45.1 leukocyte antigen). Four types of chimeras were generated: WT→WT (WT cells expressing CD45.1 into wild‐type mice expressing CD45.2); WT→IGF2BP2^−/−^ (wild‐type cells expressing CD45.1 into IGF2BP2^−/−^ mice expressing CD45.2); IGF2BP2^−/−^ → IGF2BP2^−/−^ (IGF2BP2^−/−^ cells expressing CD45.1 into IGF2BP2^−/−^ mice expressing CD45.2); and IGF2BP2^−/−^ → WT (IGF2BP2^−/−^ cells expressing CD45.1 into wild‐type mice expressing CD45.2). The level of cell engraftment was verified after 7 weeks by measuring the cell surface expression of CD45.1 and CD45.2 in blood, as described previously in this work. Colitis and asthma model was subsequently induced with DSS or Cockroach allergen.

##### iRIP‐seq

BMDMs were irradiated once for 400 mJ cm^−2^ and lysed in ice‐cold wash buffer. Cell lysis was performed in cold Wash buffer (1 × PBS, 0.1% SDS, 0.5% tergitol‐type (NP‐40), and 0.5% sodium deoxycholate) supplemented with a 200 U mL^−1^ RNase inhibitor (Takara) and protease inhibitor cocktail (Roche) and incubate on ice for 30 min. Clear cell lysate by centrifugation at 10 000 rpm for 10 min at 4 °C. Add RNA qualified (RQ) I (Promega, 1 U µL^−1^) to a final concentration of 1 U µL^−1^ and incubate in a water bath for 30 min at 37 °C. Immediately afterward, a stop solution was added to the lysates to quench DNase. The mixture was then vibrated vigorously and centrifuged at 13 000 x *g* at 4 °C for 20 min to remove cell debris. Then RNA digestion by MNase (Thermo Scientific) was performed.

For immunoprecipitation, the supernatant was incubated overnight at 4 °C with 10 µg IGF2BP2‐antibody (proteintech: 11601‐1‐AP) and control IgG‐antibody (CST: 2729s). The immunuprecipitates were further incubated with protein A/G Dynabeads (Thermo Scientific) for 2 h at 4 °C. After applying to magnet and removing the supernatants, the beads were sequentially washed with lysis buffer, high‐salt buffer (250 × 10^−3^ m Tris 7.4, 750 × 10^−3^ m NaCl, 10 × 10^−3^ m ethylenediaminetetraacetic acid (EDTA), 0.1% SDS, 0.5% NP‐40, and 0.5 deoxycholate), and T4 polynucleotide kinase (PNK) buffer (50 × 10^−3^ m Tris, 20 × 10^−3^ m ethylene glycol‐bis(β‐aminoethyl ether)‐N,N,N',N'‐tetraacetic acid (EGTA), and 0.5% NP‐40) for two times, respectively. Resuspend the beads in of Elution buffer (50 × 10^−9^ m Tris 8.0, 10 × 10^−3^ m EDTA and 1% SDS). Incubate the suspension for 20 min in a heat block at 70 °C to release the immunoprecipitated RNA binding protein (RBP) with crosslinked RNA and vortex. Remove the magnetic beads on the separator and transfer the supernatant to a clean 1.5 mL microfuge tube. Add Proteinase K (Roche) into the 1% input (without immunoprecipitated) and immunoprecipitated RBP with crosslinked RNA, with final concentration of 1.2 mg mL^−1^. Incubate for 120 min at 55 °C. The RNA was purified with Trizol reagent (Life technologies). cDNA libraries were prepared with KAPA RNA Hyper Prep Kit (KAPA, KK8541) according to the manufacturer's procedure. For high‐throughput sequencing, the libraries were prepared following the manufacturer's instructions and applied to Illumina NovaSeq 6000 system for 150 nt paired‐end sequencing.

##### RNA Pull Down Assay

Single‐stranded RNA containing methylated or unmethylated adenosine were synthesized by Biosune Biotechnology (shanghai) Co., Ltd and listed in Table S1 in the Supporting Information. PPAR*γ* or TSC1 RNA were desthiobiotin‐labeled by using Pierce RNA 3′ End Desthiobiotinylation Kit (20163, Thermo Scientific) and RNA pull‐down assays were carried out as the Pierce Magnetic RNA‐Protein Pull‐Down Kit (20164, Thermo Scientific) described. Briefly, up to 50 pmol of RNA was denatured at 85 °C for 5 min and biotin‐labeled by T4 RNA ligase. Then, biotin labeled nucleic acid incubated with 50 µL of streptavidin beads and 2 mg of BMDMs protein lysates. Finally, the eluted RNA‐binding protein complexes were boiled and assay with anti‐IGF2BP2 antibody.

##### Statistical Analysis

The statistical significance of differences in groups were performed by using GraphPad Prism 8.0 (GraphPad Software). All data and error bars are presented as the mean ± SEM (standard error of the mean) and based on experiments performed at least in triplicate. Data points were gathered such that significant variations were able to be observed in the data, *n* = 3–10 depending on the condition being presented. The two‐way ANOVA test was used to compare the mean of a continuous variable between two samples and Unpaired Student's *t*‐test was used to comparison between two groups. *P* < 0.05 was considered to be statistically significant.

## Conflict of Interest

The authors declare no conflict of interest.

## Author Contribution

X.W. performed research, analyzed the data, and wrote the paper. Y.J. performed research and analyzed the data. R.L. and J.Z. performed research. P.F. provided materials. G.L. provided intellectual input. Y.X. and Y.W. performed the iRIP‐seq and analyzed the data. C.J. funded the work. D.C. was responsible for the design of the study, interpretation of data, and writing of the paper. J.L. conceptualized the project and funded the work.

## Supporting information

Supporting Information

Supplemental Table 1

Supplemental Table 2

Supplemental Table 3

## Data Availability

The data that support the findings of this study are available in the Supporting Information of this article. The iRIP‐seq data that support the findings of this study are openly available in [Gene Expression Omnibus. GEO] at [https://www.ncbi.nlm.nih.gov/geo/query/acc.cgi?acc=GSE162128], reference number [GSE162128].
