# Supplementary material for: The m6A Reader IGF2BP2 Regulates Macrophage Phenotypic Activation and Inflammatory Diseases by Stabilizing TSC1 and PPARγ
Source: Adv Sci (Weinh). 2021 May 3;8(13):2100209. doi: 10.1002/advs.202100209 (PMC8261491; doi:10.1002/advs.202100209)
Supplement: Supplementary file 1 — Supporting Information [file ADVS-8-2100209-s001.pdf]

© 2021 Wiley-VCH GmbH

ADVANCED SCIENCE
Open Access

Supporting Information

for *Adv. Sci.,* DOI: 10.1002/advs.202100209

The m6A reader IGF2BP2 regulates macrophage phenotypic activation and inflammatory diseases by stabilizing TSC1 and PPARγ

Xia Wang[1], Yuge Ji[1], Panpan Feng[2], Rucheng Liu[1], Guosheng Li[2], Junjie Zheng[1], Yaqiang Xue[3], Yaxun Wei[4], Chunyan Ji[2], Dawei Chen[5], Jingxin Li[1]**

Supplementary Figures and supplementary figures legends

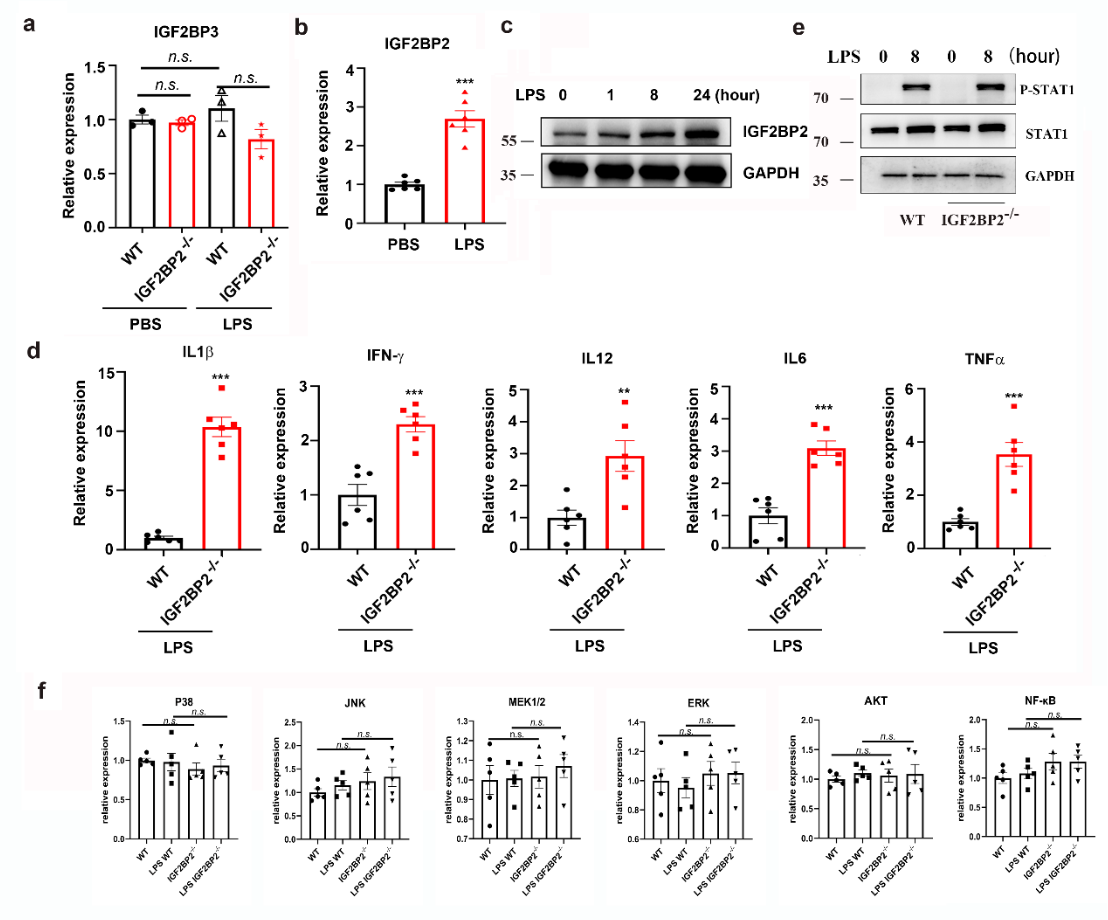

Figure S1. IGF2BP2 deficient peritoneal macrophages exaggerate inflammatory response to LPS.

(a) IGF2BP3 mRNA expression after LPS treatment for 24 hours; n = 3. (b) IGF2BP2 mRNA expression after LPS treatment for 24 hours; n = 6. (C) IGF2BP2 protein expression after LPS treatment at indicated time points; n = 4. (d) Expression of IL1β, IFN-γ, IL12, IL6, and TNF-α in the WT and IGF2BP2$^{-/-}$ peritoneal macrophages were determined by real-time PCR after LPS stimulation for 24 hours; n = 6. (e) Immunoblotting of P-STAT1and STAT1 after LPS treatment for 8 hours. (f) The total mRNA level of relative signal pathway was checked by RT-qPCR. n = 5. Data were shown as mean ± SEM. n.s. no significant, **p < 0.01, ***p < 0.001 versus the WT group by two-tailed unpaired Student’s t-test (b, d) or two-way ANOVA (a, f).

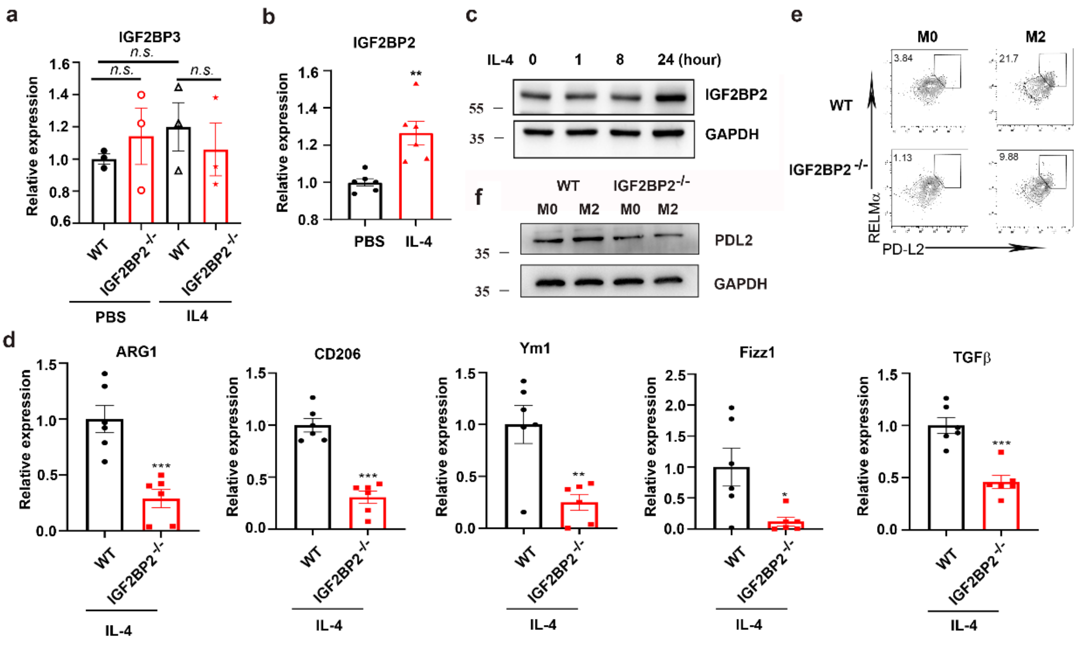

Figure S2. IGF2BP2$^{-/-}$ peritoneal macrophages exhibit defective anti-inflammatory responses to IL4.

IGF2BP3 mRNA expression after IL4 treatment for 24 hours; n = 3. (b) IGF2BP2 mRNA expression after IL4 treatment for 24 hours; n = 6. (c) IGF2BP2 protein expression after IL4 treatment at indicated time points; n = 4. (d) Expressions of ARG1, Ym1, Fizz1, CD206, and TGF-β in the WT and IGF2BP2$^{-/-}$ peritoneal macrophages were determined by real-time PCR after IL4 stimulation for 24 hours; n = 6. (e) Representative flow analysis of PD-L2 and Relm-α expression on the F4/80^{+}CD11b^{+} population after IL4 treatment BMDMs for 24 hours. Percentages are shown in the top left corner. (f) The expression of PDL2 was checked by western blot analysis. Data were shown as mean ± SEM. n.s. no significant, **p < 0.01, ***p < 0.001 versus the WT group by two-tailed unpaired Student's t-test (b, d) or two-way ANOVA (a).

a

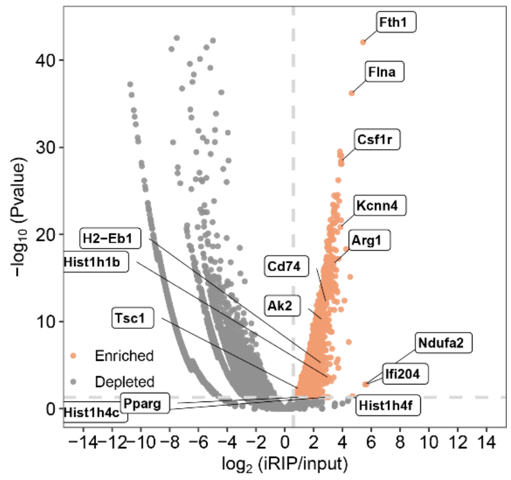

b

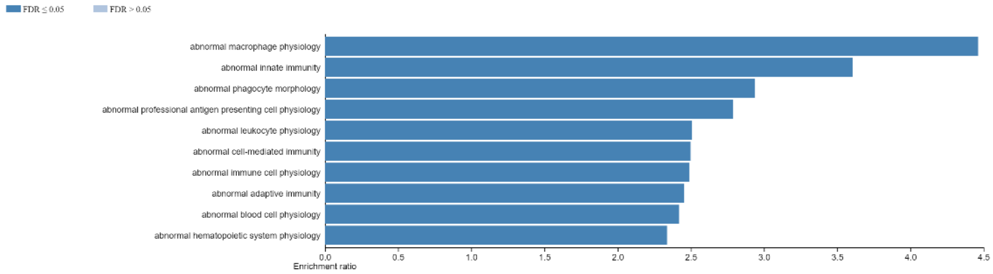

Figure S3 iRIP-seq analysis of IGF2BP2-bound mRNAs from BMDMs.

(a) Volcano plot shows IGF2BP2-bound mRNAs. x axis shows log-fold change and y axis shows p value for corresponding binding-mRNA. Representative enriched mRNAs are highlighted in orange. (b) GO analysis of mammalian phenotype ontology.

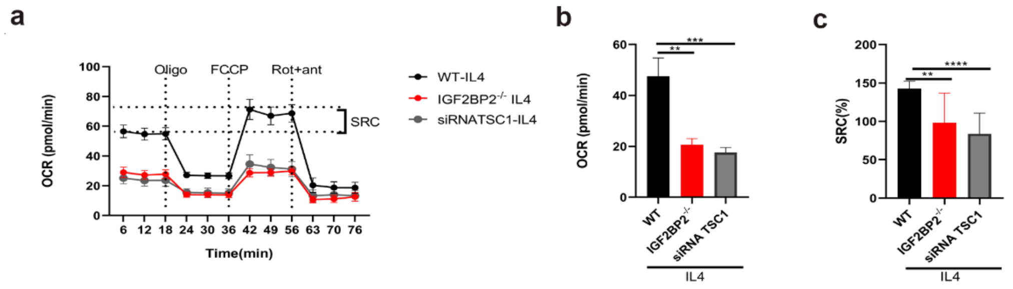

Figure S4. M2 metabolomic reprograming regulated by IGF2BP2 is linked to TSC1.

(a)Real-time changes in OCR were analyzed in WT, IGF2BP2$^{-/-}$ and siRNATSC1 BMDMs cultured for 8 hours in IL-4 by a Seahorse analyzer following sequential treatment with oligomycin, FCCP and rotenone plus antimycin. b, c) OCR (b) and SRC (c) of BMDMs after culture in IL-4 for 8 hours; n = 3. Data are shown as mean ± SEM. **p < 0.01 versus the WT BMDMs by two-way ANOVA .

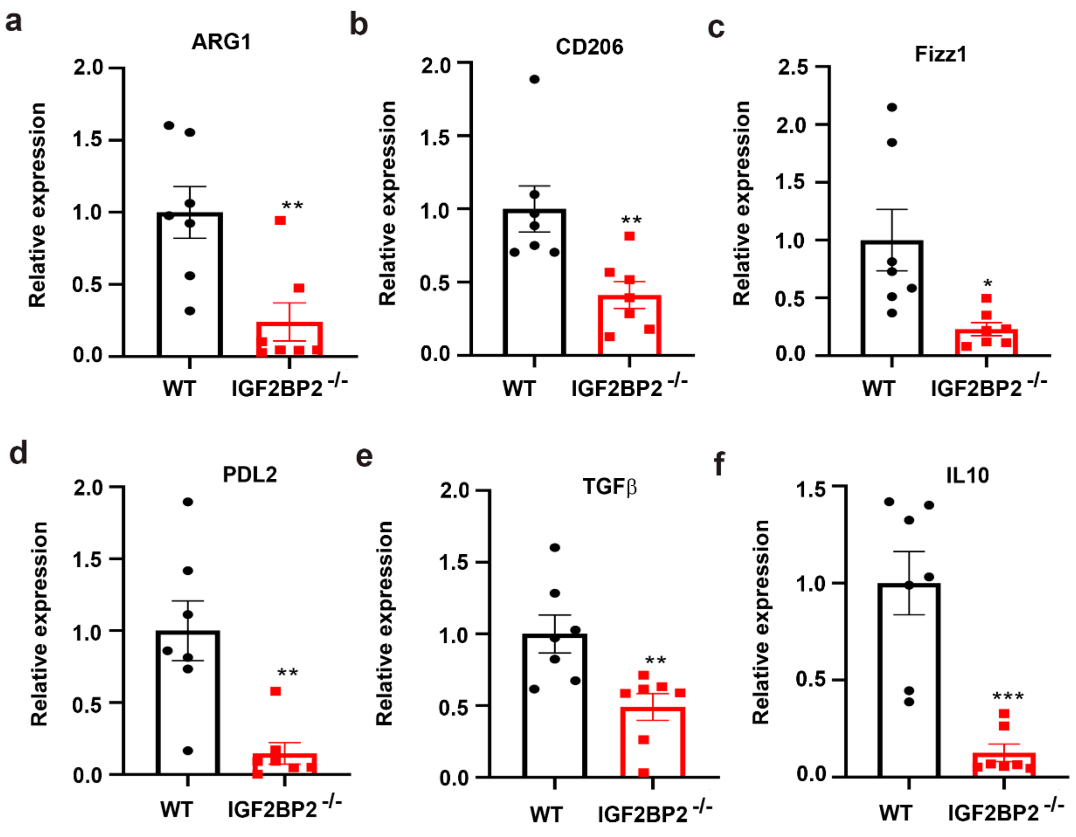

Figure S5. M2 polarization is impaired in chitin injected IGF2BP2$^{-/-}$ mice

a-f) Anti-inflammatory marker genes expression in peritoneal exudate cells (PECs) from wild-type and IGF2BP2$^{-/-}$ mice determined by RT-qPCR after chitin injection; n=7. Data were shown as mean ± SEM. **$p < 0.01$, ***$p < 0.001$ versus the WT group by two-tailed unpaired Student's t-test.

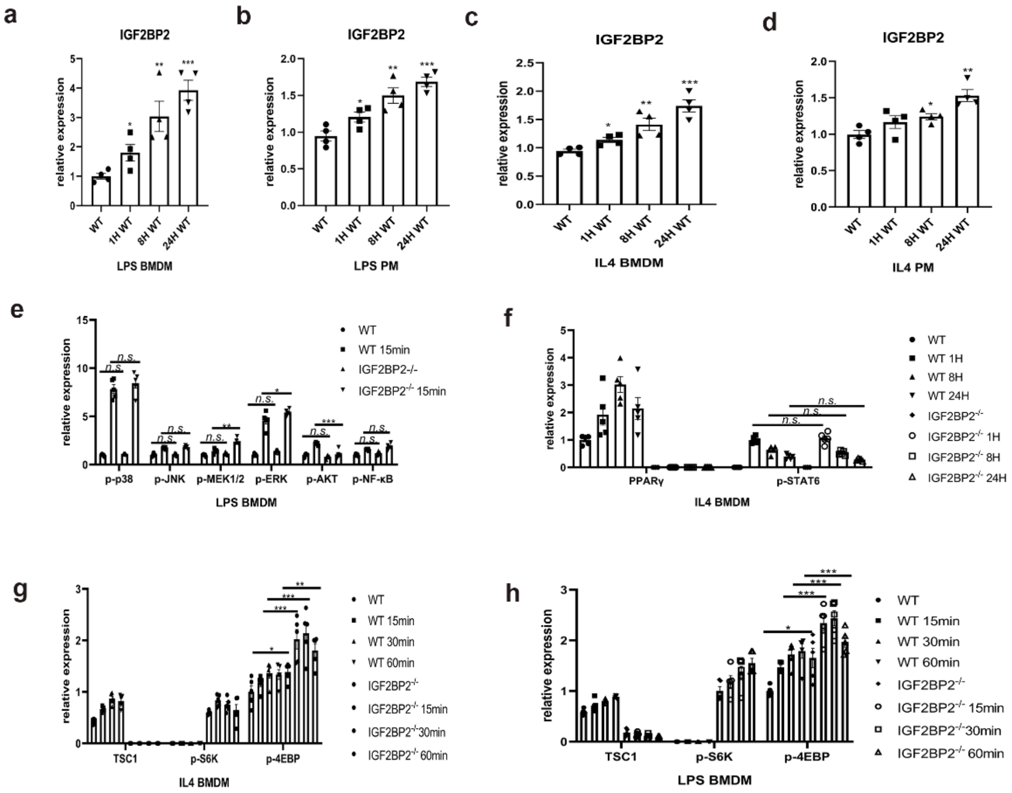

Figure S6. Histogram of protein normalization.

a-h) Immunoblot analysis of the indicated proteins in BMDMs (a, c, e, f, g, h) or PMs (b, d) prepared from WT and IGF2BP2$^{-/-}$ mice, followed by densitometric analysis of the blots; Data are presented as means ± SEM. *P < 0.05; **p < 0.01, ***p < 0.001 versus the WT group by two-tailed unpaired Student's t-test (a-d) or two-way ANOVA (e-h).
